# In Silico Analysis of Metabolites from Peruvian Native Plants as Potential Therapeutics against Alzheimer’s Disease

**DOI:** 10.3390/molecules27030918

**Published:** 2022-01-28

**Authors:** Luis Daniel Goyzueta-Mamani, Haruna Luz Barazorda-Ccahuana, Miguel Angel Chávez-Fumagalli, Karla Lucia F. Alvarez, Jorge Alberto Aguilar-Pineda, Karin Jannet Vera-Lopez, Christian Lacks Lino Cardenas

**Affiliations:** 1 Laboratory of Genomics and Neurovascular Diseases, Universidad Católica de Santa María, Urb. San José s/n—Umacollo, Arequipa 04000, Peru; mchavezf@ucsm.edu.pe (M.A.C.-F.); kalvarezf@ucsm.edu.pe (K.L.F.A.); jaguilar@ucsm.edu.pe (J.A.A.-P.); kvera@ucsm.edu.pe (K.J.V.-L.); 2 Vicerrectorado de Investigación, Universidad Católica de Santa María, Urb. San José s/n—Umacollo, Arequipa 04000, Peru; hbarazorda@ucsm.edu.pe; 3 Cardiovascular Research Center, Massachusetts General Hospital, Boston, MA 02114, USA

**Keywords:** in silico, Peruvian native plants, Alzheimer’s disease, polypharmacology, floribundic acid, rutin, brassicasterol, Peru

## Abstract

Background: Despite research on the molecular bases of Alzheimer’s disease (AD), effective therapies against its progression are still needed. Recent studies have shown direct links between AD progression and neurovascular dysfunction, highlighting it as a potential target for new therapeutics development. In this work, we screened and evaluated the inhibitory effect of natural compounds from native Peruvian plants against tau protein, amyloid beta, and angiotensin II type 1 receptor (AT1R) pathologic AD markers. Methods: We applied in silico analysis, such as virtual screening, molecular docking, molecular dynamics simulation (MD), and MM/GBSA estimation, to identify metabolites from Peruvian plants with inhibitory properties, and compared them to nicotinamide, telmisartan, and grapeseed extract drugs in clinical trials. Results: Our results demonstrated the increased bioactivity of three plants’ metabolites against tau protein, amyloid beta, and AT1R. The MD simulations indicated the stability of the AT1R:floribundic acid, amyloid beta:rutin, and tau:brassicasterol systems. A polypharmaceutical potential was observed for rutin due to its high affinity to AT1R, amyloid beta, and tau. The metabolite floribundic acid showed bioactivity against the AT1R and tau, and the metabolite brassicasterol showed bioactivity against the amyloid beta and tau. Conclusions: This study has identified molecules from native Peruvian plants that have the potential to bind three pathologic markers of AD.

## 1. Introduction

Alzheimer’s disease (AD) is a chronic neurodegenerative disorder characterized by the progressive manifestations of disturbances on rational thinking, memory loss, cognitive decline, and mood changes [1,2,3]. These clinical manifestations are consequences of the formation of neurofibrillary tangles, senile plaques, glial cell activation, and cerebrovascular dysregulation [4]. The importance of understanding the mechanisms of cerebrovascular alterations and their relation to AD has gained more attention, since cerebrovascular dysfunction can cause the degenerative processes of smooth muscle cells, astrocytes, pericytes, and endothelial cells [5,6,7,8]. Furthermore, several studies have demonstrated the reduced resting cerebral blood flow, low vasoreactivity, and neurovascular coupling dysregulation in AD patients [9,10,11], and that more than 50% of AD-diagnosed patients also have a cerebrovascular lesion.

Besides the tau protein and amyloid beta, the constant search for novel molecular targets has implicated the renin–angiotensin system (RAS), which is involved in cerebrovascular functions in AD patients [12]. The RAS is a complex enzymatic pathway that regulates fluid homeostasis, blood pressure, and cognitive responses [13]. A key enzyme of this complex is the angiotensin II peptide, a vasoconstrictor that induces hypertensive responses [12,14]. The angiotensin peptides develop their function through receptors, such as the angiotensin II type I receptor (AT1R), angiotensin II type 2 receptor (AT2R), and angiotensin II type 4 receptor (AT4R) [15,16]. The AT1R, located on neurons, astrocytes, and microglia [17], has the function of NADPH oxidase complex activation, which leads to superoxide formation, vasoconstriction, proinflammatory, and pro-oxidative effects [15,16]. For this reason, research on AT1R blockers (ARBs) has increased in the last decade due to the findings on the increment of ACE activity in AD patients.

Extensive research on natural sources has been carried out as potential options for finding new therapeutic AD treatment interventions [18]. In this context, the ethnopharmacological uses of medicinal plants for treating hypertension have been documented in North America, Europe, Asia, and Africa [19]. Plants from the species *Guazuma ulnifolia* (from Central America) [20], *Radix Astragali Mongolici, Salvia miltiorrhiza bunge, Flos Lonicerae, Scrophularia, Radix Aconiti Lateralis, Preparata,* and *Radix glycyrrhizae* (from Asia) showed a blocking effect on AT1R activity, mainly due to the presence of polyphenols, saponins, and flavonoids [21,22]. More recently, the phytochemicals gastrodin and silibinin, found in *Gastrodia elata Blume* (Tianma) and *Cirsium japonicum* (Japanese Thistle), respectively, showed an antagonist effect for AT1R [23,24,25]. Moreover, the phytochemicals quercetin and chlorogenic acid, components of *Campomanesia xanthocarpa*, a Brazilian endemic species, could act as an AT1R blocker, and were proposed as a preventive agent for high blood pressure [26,27]. The number of Peruvian medicinal plants has been calculated to be approximately 25,000 species [28,29], and their ethnopharmacological uses, to treat or relieve the symptoms of vascular diseases or dementia-associated diseases, have been described [30]. For example, medicinal plants such as *Lepidium meyenii* (maca) have been studied for their neuroprotective and antihypertensive properties, due to their bioactive metabolites targeting the ACE receptor [31,32,33]. Another plant is the *Uncaria* genus (cat’s claw), which has been studied for its catecholamines metabolites that regulate blood pressure and heart rate [34,35], and the *Zea mays* (purple corn), which are rich in flavonoids, such as morin, quercetin, and kaempferol, that have demonstrated a neuroprotective and vasodilatory effect via the nitric oxide-cyclic guanosine monophosphate-protein kinase G (NO–cGMP–PKG) pathway [36,37]. However, the knowledge of the beneficial effects of active compounds isolated from Peruvian medicinal plants is still limited [38].

In diseases that are multifactorial in origin, such as AD, a new perspective towards drug discovery and development includes moving from target-specific to multi-target drugs [39]. Hence, computational polypharmacology has become a valuable support [40], since it makes it possible to simulate and screen thousands of molecules that can bind to several targets. In this way, a set of ligands against a set of targets can be simulated, anticipating the potential selectivity, multi-target activities, and optimization of screening processes [41].

In this work, based on an in silico and computer-aided drug screening, we aimed to show the therapeutic potential of three metabolites (rutin, brassicasterol, and floribundic acid) derived from Peruvian plants for the possible treatment of patients with Alzheimer’s disease. Remarkably, we found that rutin showed polypharmacological bioactivity against tau, amyloid beta, and AT1R.

## 2. Results

### 2.1. Literature Research

The natural compounds literature from Peruvian plants were retrieved from the PubMed database (https://pubmed.ncbi.nlm.nih.gov, accessed on 15 July 2021). Our data mining has identified 84 metabolites from Peruvian plants previously characterized biochemically from 1970 to 2020 (Tables S1–S6). Then, we filtered these metabolites by their neuroprotective, antihypertensive and antioxidant properties, finding the following native plants: *Smallanthus sonchifolius* (Yacon) of the family Asteraceae, showing that the antihypertensive and anti-inflammatory properties of the leaf extract on LPS-stimulated mouse microglial cells in vitro [42] prevented the deposition of amyloid plaques and neurotoxicity because of its antioxidant effect on the hippocampus [43]. *Lepidium meyenii* (Maca) is a hypocotyl of the family Brassicaceae with neuroprotective effects due to the inhibition of acetylcholinesterase (AChE) activity, improving scopolamine-induced memory deficits [38,44]. Secondary metabolites, such as macamides have shown a reduction of Mn-induced mitochondrial toxicity in glioblastoma U/87 MG cells, regulating inflammation, and glucose homeostasis, hence beneficial for AD treatment [45,46].

*Croton lechleri* (Sangre de Drago), of the family Euphorbiaceae, a sap rich in phenolic compounds with the ability to reduce ROS formation, LDL oxidations, and albumin glycation, thereby reducing the risk of vascular diseases [47,48]. *Uncaria tomentosa* (Uña de Gato or Cat’s claw) is a vine of the Rubiaceae family with antimutagenic and neuroprotective properties. The polyphenols, sterols, and alkaloids present in Cat’s Claw have shown a positive effect in cholinergic dysfunction, inhibiting AChE activity and DNA repairment, avoiding oxidative stress and inflammation, and thus, cardiovascular and autoimmune diseases [49,50,51]. *Physalis peruviana* (Aguaymanto) is a berry of the Solanaceae family with anti-inflammatory and cytoprotective properties in astrocytic cells (T98G) exposed to neurotoxic stimuli, preserving mitochondrial functions and nuclei damage [52,53]. *Minthostachys mollis* (Muña) of the Lamiaceae family is rich in flavonoids that inhibit COX1 and COX2 enzymatic production, hence avoiding the proinflammatory process. According to Benites et al., the essential oil of this species has a potential cytotoxic effect against cancer cells (T24, DU-145, and MCF-7) [54,55].

### 2.2. Docking Procedure and Virtual Screening

**Figure 1:**
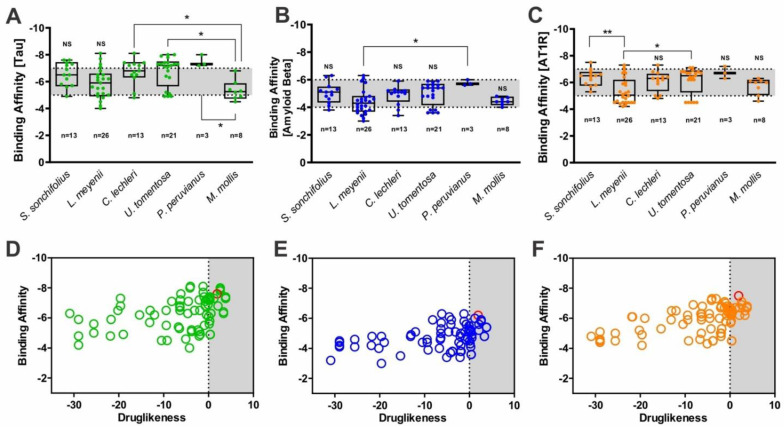
Binding affinities of molecules screened from *Smallanthus sonchifolius, Lepidium meyenii, Croton lechieri, Uncaria tomentosa, Physialis perivianus*, and *Minthostachys mollis* against: (**A**) tau peptide, (**B**) amyloid beta (1–40), and (**C**) AT1R. NS: not significant. * *p* < 0.05 and ** *p* < 0.0.1 significance among groups. Box plots with minimum and maximum values of binding affinities. Scatter plot showing binding affinity versus drug-likeness score for: (**D**) tau, (**E**) amyloid beta (1–40), and (**F**) AT1R.

Virtual screening was performed to screening potential molecules against tau, amyloid beta, and AT1R (Figure 1 and Tables S7–S9). Among these molecules, we selected the top three with the lowest binding affinity energy for our study: rutin (effective against the three receptors), brassicasterol (effective against tau and amyloid beta), and floribundic acid (effective against tau and AT1R). Figure 1 also shows the drug-likeness score calculation of the complete set of molecules against the three targets using OSIRIS DataWarrior, where more potential molecules are represented for possibly further analysis.

In parallel, studied compounds from ongoing clinical and preclinical studies (phase II and III) were also analyzed and used as controls (Table 1). We chose one compound per receptor as control: nicotinamide for tau, grapeseed extract for amyloid beta, and telmisartan for AT1R.

### 2.3. ADME/TOX Analysis

The ADME/TOX study was performed to investigate and evaluate the therapeutic potential of metabolites derived from Peruvian plants to predict their metabolism in the human body. In Table 2, the toxicity assessment of the chosen compounds was also analyzed to evaluate their hepatotoxicity, carcinogenicity, mutagenicity, and cytotoxicity. Most compounds showed a favorable effect in this screening, except floribundic acid and kaurenoic acid, which showed a cytotoxic and hepatotoxic effect, respectively.

### 2.4. Molecular Dynamics Simulations and Molecular Mechanics Generalized Born Surface Area Calculations

**Figure 2:**
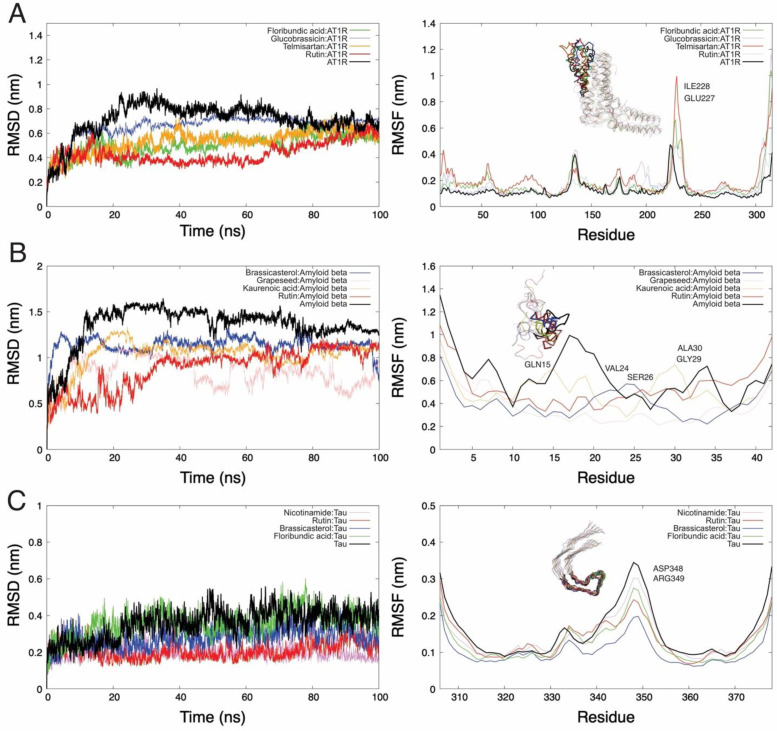
Representation of DM trajectories over 100 ns for AT1R, amyloid beta, and tau. The figures on the right side are the RMSD plots, and those on the left side are the RMSF plots of the backbone *per residue*. (**A**) The tendency of the RMSD plot for the AT1R–rutin system shows that the protein does not constantly deviate from its original conformation even though this compound generates the largest fluctuations in AT1R (RMSF analysis); (**B**) the RMSD plot for amyloid beta in its native state (black line) shows a high structural instability due to the size and lack of other secondary conformations. However, the coupled systems reduce this deviation; (**C**) tau with ligands shows a tendency of high conformational stability.

The active sites or regions of the receptors against the ligands were determined using the PATCHDOCK server (http://bioinfo3d.cs.tau.ac.il/PatchDock/, accessed on 10 August 2021). The snapshot obtained from 100 ns of NPT simulation was used for the thermodynamics parameter calculations. The root mean squared deviation (RMSD) and root mean squared fluctuations (RMSF) per residue of the backbone were analyzed. The structure variation was calculated by RMSD values of protein:ligand from 0 to 100 ns, reaching stability at 70 ns (Figure 2). We can see in Figure 2A that the RMSD values for AT1R receptors were the following: native receptor (0.71 nm), rutin (0.43 nm), floribundic acid (0.50 nm), telmisartan (0.53 nm), and glucobrassicin (0.66 nm). On the other hand, in the RMSF per residue results, the AT1R:rutin system shows high fluctuations in loop regions (region highlighted from protein AT1R in Figure 2A), with a high peak of 0.7 nm.

When we looked at the RMSD plot of amyloid beta (Figure 2B), we noticed a significant deviation in the RMSD trend in all systems analyzed. Indeed, the RMSD average values for the native receptor (1.33 nm), grapeseed extract (0.84 nm), rutin (0.88 nm), kaurenoic acid (1.03 nm), and brassicasterol (1.14 nm) were obtained. This was caused by the lack of secondary regions in the peptides. However, when it is docked, the fluctuations are reduced (RMSF plot from Figure 2B). Figure 2C shows the RMSD graph concerning the initial structure of tau, where the comparison of the average RMSDs between the tau receptor is less than 0.36 nm, and an average RMSD for tau–rutin and tau–nicotinamide is equal to 0.26 nm. At the same time, the RMSF *per residue* of the tau indicates the reduction of fluctuations in coupled systems (RMSF plot from Figure 2C).

The molecular mechanics generalized born surface area (MM/GBSA) binding free energy estimation was carried out considering the last 500 snapshots from MD simulations of receptor–ligand systems.

Likewise, the generalized born (GB) model and solvent accessibility methods are used for the MM/GBSA calculation. The results obtained of this estimation are shown in Table 3, in which among the ligand–receptor systems, the best option for amyloid beta, tau, and AT1R was rutin (48.05 ± 7.0 kcal·mol^−1^), brassicasterol (−38.05 ± 3.5 kcal·mol^−1^), and floribundic acid (58.98 ± 4.4 kcal·mol^−1^), respectively. The second-best option, and shared among the three receptors, were rutin against amyloid beta, tau, and AT1R, in which the binding energy was found to be equal to 48.05 ± 7.0 kcal·mol^−1^, −27.25 ± 6.1 kcal·mol^−1^, and −39.56 ± 3.3 kcal·mol^−1^, respectively. There is significant energy contribution by the Van der Waals energies in the systems regarding electrostatic and generalized born energies. Substances under clinical trials were run in order to be used as controls, acting as references point to evaluate the potential anti-Alzheimer effect of the Peruvian natural plants.

Thereof, based on these calculations, the ligands rutin for amyloid beta, brassicasterol for tau, and floribundic acid for AT1R were deduced with considerable binding affinity by comparison to their control reference, which was grapeseed extract, nicotinamide, and telmisartan, respectively.

## 3. Discussion

For the last three decades, over 900 billion USD has been invested in drug development and clinical trials with no proven effective benefit for treating patients with AD [56]. Multiple studies have recently demonstrated a strong relationship between the pathogenesis of AD and some vascular-associated diseases, including atherosclerosis and hypertension [57,58]. Moreover, Aguilar et al., 2021, have shown that vascular smooth muscle cells contribute to the neuroinflammation and tau hyperphosphorylation in the early and late stages of the disease [6]. These findings identify the vascular tissue as a novel therapeutic target for the development of drugs to reduce the impact of vascular dysfunction on the initiation and progression of AD. In this context, the neuroprotective effects of AT1R blockers (Losartan and Resveratrol) indicate that RAS components can be plausible targets to develop pharmacological innovations for AD [59,60].

For these reasons, the research for new compounds with neurovascular or anti-neurodegenerative bioactivity is gaining considerable attention in the field [61]. In this regard, a total of 94 options between drugs and antibodies are in clinical trials (phases II and III) [62]. Among those compounds, some natural products, such as grapeseed extract, nicotinamide, nicotine, saffron, colostrinin, and others, are considered as promising therapeutic candidates [63,64,65]. Furthermore, in vivo experiments using animal models of dementia and AD have been used to evaluate the potential therapeutic potential of other plant-based molecules, such as curcumin, quercetin, resveratrol, piperine, and epigallocatechin gallate.

Peru is known worldwide for its incredible phytogenic biodiversity, in which ~10% of the world’s flora grows [37]. Polypharmacology is emerging as a new paradigm to treat complex diseases by regulating multiple targets to achieve desired physiological responses [66]. Additionally, a multi-target drug can decrease the risk of drug−drug interactions and diminish the number of pharmacokinetic and safety profile tests [67]. The ethnopharmacological knowledge of the Peruvian population helped to identify and categorize more than 5000 Peruvian plants with multiple pharmacological effects.

In this sense, we aimed to characterize the polypharmacological properties of metabolites from Peruvian plants known by their neuroprotective and vasoactive properties and thus identify potential therapeutic candidates for treating patients with AD.

Based on our in silico and computer-aided drug screening, we found remarkable bioactivity against three pathologic markers of AD, including tau protein, amyloid beta and AT1R. Our results demonstrated the highest binding energy for the systems as follows: AT1R:floribundic acid (a compound present in *S. sonchilofolius*), amyloid beta:brassicasterol (a compound present in *L. meyenii*), and tau–rutin (a compound present in *C. lechleri*). Interestingly, the rutin molecule has shown strong polypharmacological activity against all three pathogenic markers, brassicasterol against tau and amyloid beta, and floribundic acid against tau and AT1R. It is worth mentioning that the metabolite rutin showed a higher affinity for tau protein, amyloid beta, and AT1R compared to the controls, drugs in clinical trials, such as nicotinamide, grapeseed extract, and telmisartan, respectively. In conclusion, we identified three metabolites with the potential to bind three pathologic markers of AD and inform the development of future therapies against AD and other neurodegenerative diseases.

In addition, the beneficial effects of these metabolites have been reported by different authors. Rutin has shown an inhibitory effect on α-glucosidase, effective for the treatment of diabetes [68], an antiviral effect on SARS-CoV-2 [69], and anti-inflammatory [70] and anticarcinogenic properties [71]. The sterol brassicasterol has shown a dual anti-infective property against herpes simplex virus type 1 and *Mycobacterium tuberculosis* [72], an antiviral effect against human adenovirus [73], and an anti-tumoral [74] and potential application as an AD biomarker [75]. For floribundic acid, besides its antioxidant effect, there is no other research based on the effects or bioactivity of this clerodane diterpenoid [76].

**Figure 3:**
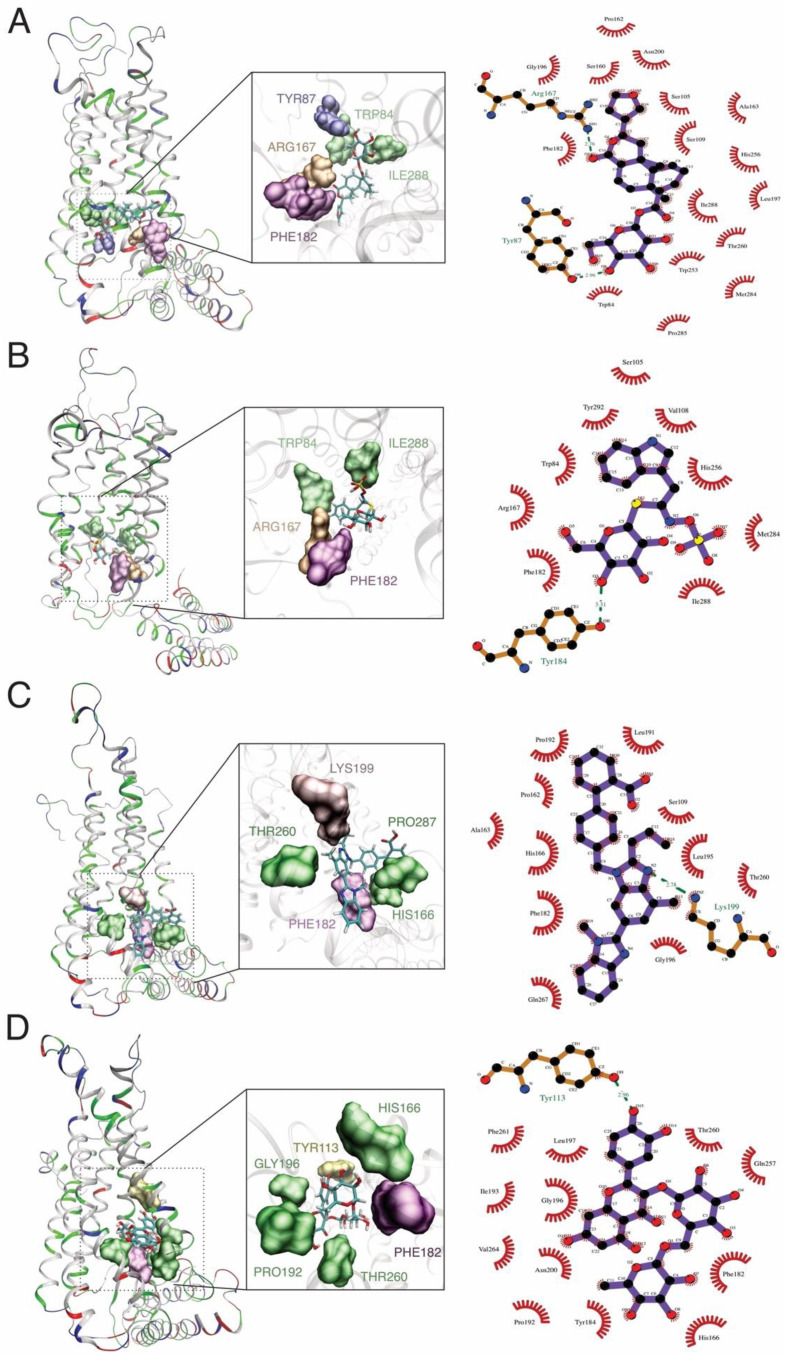
MD simulation and docking analysis shows the formation of hydrogen bond interactions between the AT1R and: (**A**) floribundic acid, (**B**) glucobrassicin, (**C**) telmisartan, and (**D**) rutin.

The binding site of the proteins is essential during the protein–ligand interaction and crucial for drug design. Our study revealed the catalytic dyad for AT1R composed of hydrogen bonds with floribundic acid (ARG167, TYR113), glucobrassicin (TYR184), rutin (TYR113) telmisartan (LYS199), and hydrophobic bonds (TYR87, TYR175, ILE288, TRP84, THR260, and PHE182 as common interacting residues (Figure 3)). Similar binding sites were observed with Olmesartan, where LYS 199, ARG167, TYR113, and TRP84 were noticed [77,78].

**Figure 4:**
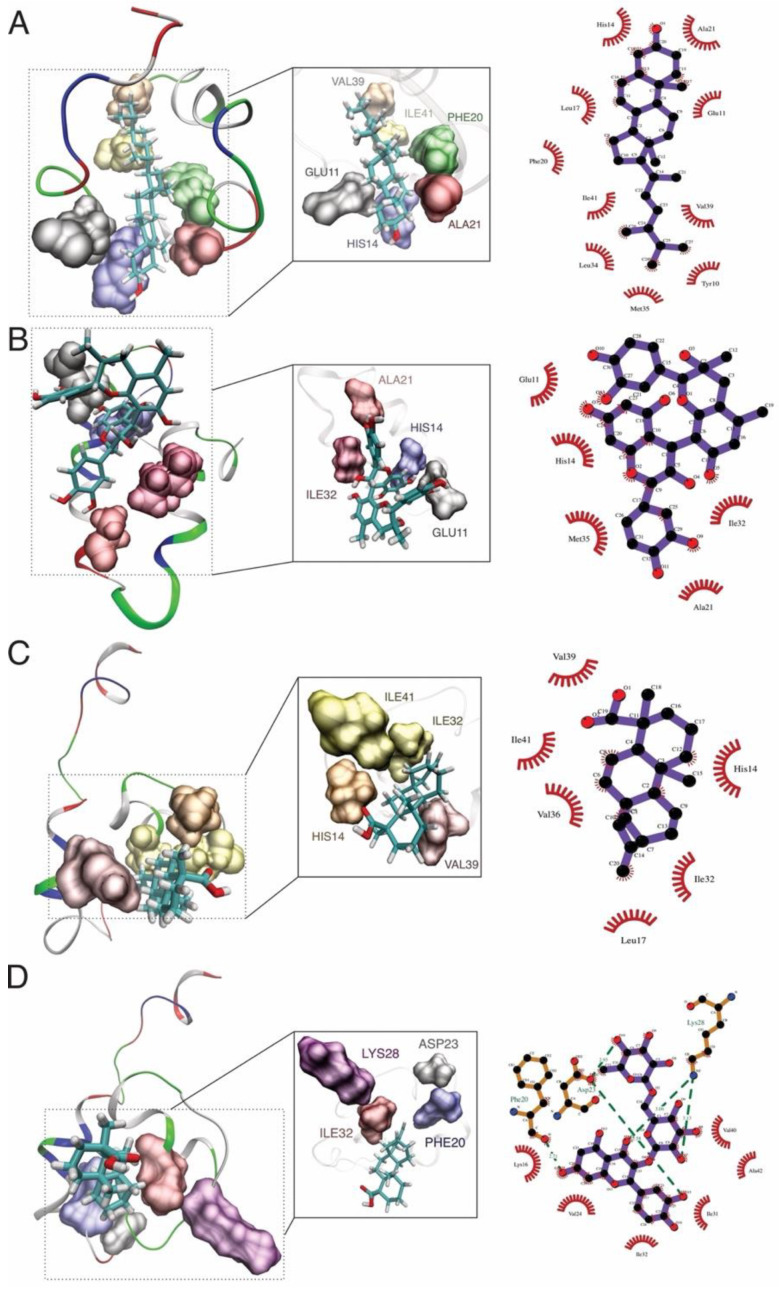
MD simulation and docking analysis shows the formation of hydrogen bond interactions between the amyloid beta and: (**A**) brassicasterol, (**B**) grapeseed extract, (**C**) kaurenoic acid, and (**D**) rutin binding interactions between the ligand and receptor.

For amyloid beta, the hydrogen bonds were observed with rutin (LYS28, ASP23, and PHE20) and hydrophobic bonds (HIS14, VAL39, ALA21, ILE32, and ILE41) as common interacting residues (Figure 4). Our results agree with other in silico evidence of the interaction between statins and amyloid beta, where authors evaluated the protective role of these drugs against amyloidogenesis and neurodegeneration, such as atorvastatin, rosuvastatin, pravastatin, and lovastatin, where ASP23, VAL39, and ALA21 residues were identified, respectively [79]. According to Castro et al., 2020, fucosterol from the algae *Sargassum horridum* showed an inhibitory aggregation effect of amyloid beta fibrils and stabilized and interacted with HIS14, PHE20, ALA21, ILE32, VAL39, ILE41, and LYS28 residues [80]. The residue HIS14 was also observed in the binding site of memantine and amyloid beta [81].

**Figure 5:**
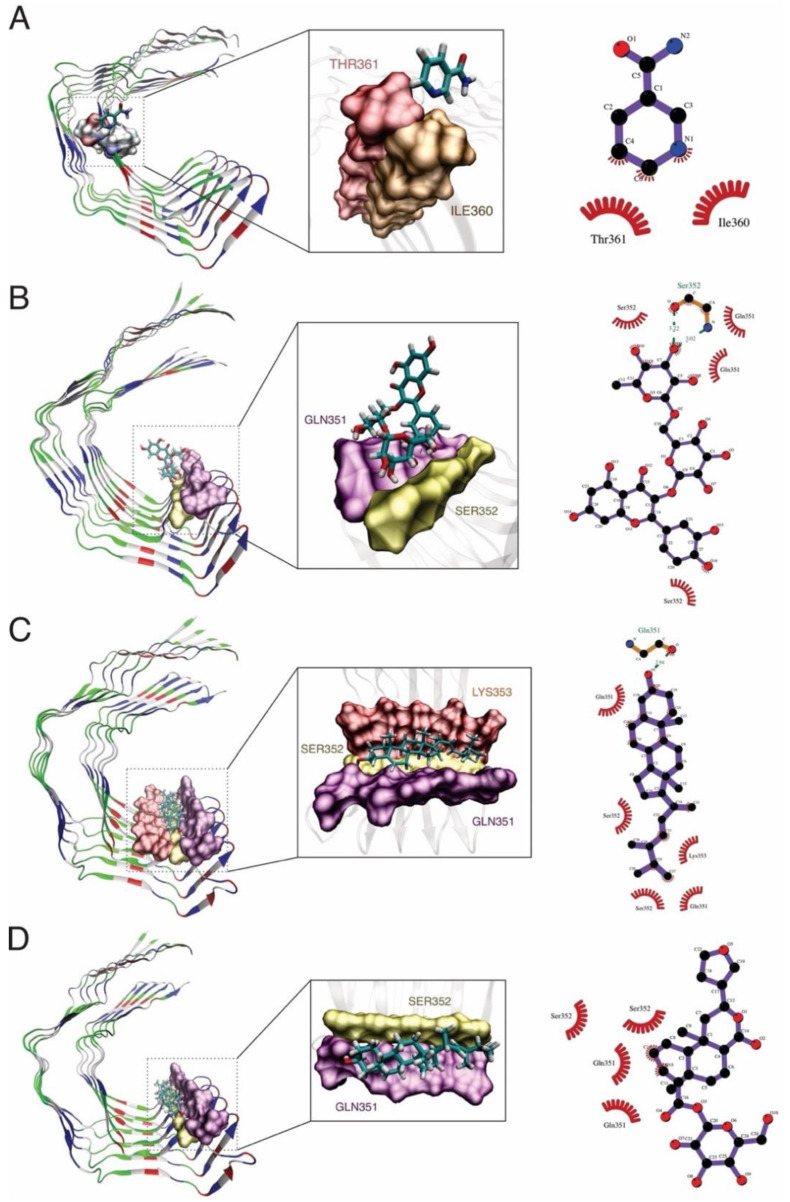
MD simulation and docking analysis shows the formation of hydrogen bond interactions between the tau protein and: (**A**) nicotinamide, (**B**) rutin, (**C**) brassicasterol, and (**D**) floribundic acid binding interactions between the ligand and receptor.

For tau, hydrogen bonds were observed with rutin (SER352), brassicasterol (GLN351), ILE360, and THR361 as common interacting residues (Figure 5). In fact, the residue GLN351 was also an active binding site for the interaction with titanium dioxide nanoparticles, where the alteration of secondary and tertiary structures of tau was aimed at to avoid further aggregation [82]. In addition, some second-generation positron emission tomography tau tracers were developed and engineered to target the active site found in our work, composed of GLN351and ILE360 [83,84].

**Figure 6:**
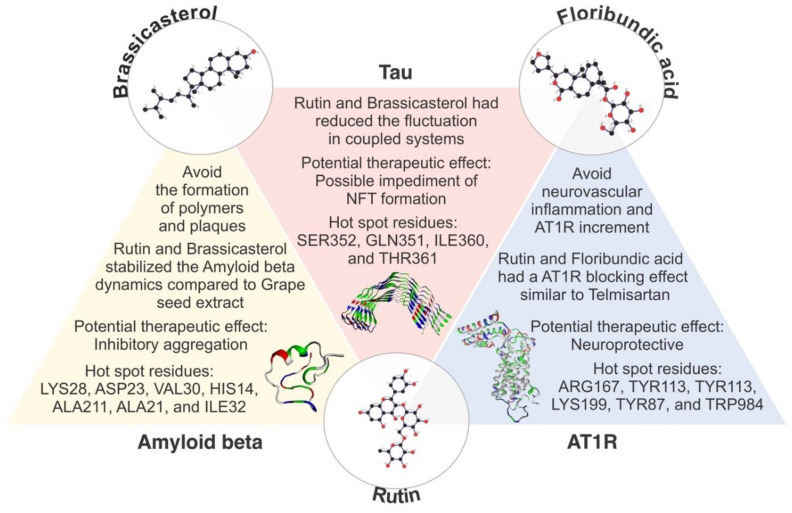
Diagram illustrating the polypharmacological activity of the metabolites rutin, brassicasterol, and floribundic acid on tau, amyloid beta, and AT1R, AD pathologic markers.

The MD simulation analyzes the stability and dynamic states of the protein–ligand systems. The protein backbone RMSD of tau, amyloid beta, and AT1R, bound with the best option of the ligands are represented in Figure 6. We observed that tau, amyloid beta, and AT1R started to stabilize and equilibrate at 60 ns, and all backbones remained stable compared to their respective controls (proteins without ligand binding). The stability of the compound related to AT1R was: rutin > floribundic acid >> telmisartan > glucobrassicin; the compound related to amyloid beta was: grapeseed extract > rutin > kaurenoic acid > brassicasterol; and the compound related to tau was: rutin > nicotinamide > brassicasterol > floribundic acid. The slight variation may be attributed to the complex conformations.

In the same figure, the RMSF analysis showed a fluctuation in the residue regions of 200–250 for AT1R, 10–35 for amyloid beta, and 340–360 for tau. The RMSF values of the systems relative to the amino acids of the complexes were evaluated to compare their flexibilities. The more fluctuated amino acids in the active sites, related to their ligands, were for AT1R, ILE228, and GLU227 attributed to the EIQKN sequence, an intracellular loop of the AT1R and a cytoplasmatic domain essential for receptor activation and G-protein selection and coupling [85]. For amyloid beta, the residues GLN15, VAL24, and SER26 were attributed to the α-helices. The ALA30 and GLY29 regions were less structured and formed an α-helix (unfolded structures). This α-helix stabilization may be crucial to prevent the formation of the insoluble β-sheet form of amyloid beta [86,87]. For tau, the amino acids the ASP348 and ARG349 residues are responsible for saline bonds formation and β-helical folding, arrangements found in the AD–tau fibrils.

Surprisingly, the molecules considered as controls, such as grapeseed extract, nicotinamide, and telmisartan for the receptors amyloid beta, tau protein, and AT1R, respectively, were not the best options with the highest binding energy [88,89].

## 4. Materials and Methods

### 4.1. Literature Search Strategy and Data Collection

The literature search strategy for collecting the Peruvian natural products and ongoing-clinical drugs was found on the National Center for Biotechnology Information (NCBI) databases, PubMed (accessed on 15 July 2021), and ChEMBL. Duplicated compounds were removed, and only registered, catalogued, and well-characterized compounds were considered for analysis.

The Medical Subject Headings (MeSH terms) were employed in the string query to improve the search accuracy. The dataset was retrieved from PubMed on 16 July 2021, based on the following search string: “Peruvian” [MeSH Terms] AND “Natural products” [MeSH Terms] AND “Alzheimer disease”.

After the first step of screening of the compounds from natural sources (*Physalis peruvianus* [90,91,92], *Minthostachys mollis* [54,93], *Uncaria tomentosa* [94], *Croton lechleri* [76,95,96], *Smallanthus sonchifolius* [97,98,99,100], and *Lepidium meyenii* [101,102]), the simplified molecular-input line-entry system (SMILE) was searched and retrieved from DrugBank (https://go.drugbank.com, accessed on 17 July 2021) or PubChem (https://pubchem.ncbi.nlm.nih.gov, accessed on 18 July 2021) servers. The physicochemical properties such as total molecular weight (MW), octanol/water partition coefficient (cLogP), number of H-bond acceptors (HBAs), number of H-bond donors (HBDs), and the molecular polar surface area (PSA) for each compound were calculated within the Osiris DataWarrior v05.02.01 software [103].

### 4.2. Docking Procedure and Virtual Screening

The FASTA sequences of the compound targets the angiotensin II type 1 receptor (AT1R) (PDB ID: 4ZUD), tau (region from the 306 to 378 residues) (PDB ID: 5O3T), and amyloid beta (PDB ID: 1IYT) were retrieved from Protein Data Bank (https://www.rcsb.org, accessed on 10 September 2021). Furthermore, the compounds were imported into OpenBabel within the Python Prescription Virtual Screening Tool (PyRx) [104] and subjected to energy minimization. PyRx performs structure-based virtual screening, applying docking simulations using the AutoDock Vina tool [105], whereas the drug targets were uploaded as macromolecules. For the analysis, the search space encompassed the whole of the modelled 3D models; the docking simulation was then run at an exhaustiveness of 8 and set to only output the lowest energy pose.

The Osiris DataWarrior software was employed to calculate the drug-likeness score of each compound; the calculation is based on a library of ~5300 substructure fragments and their associated drug-likeness scores. This library was prepared by fragmenting 3300 commercial drugs and 15,000 commercial non-drug-like Fluka compounds. Furthermore, the potential tumorigenic, mutagenic, and irritant actions of each compound were predicted by comparison to a precompiled fragment library derived from the RTECS (Registry of Toxic Effects of Chemical Substances) database [103].

### 4.3. ADME/TOX

The Lipinski rule of five (RO5) was used to evaluate compounds that violated more than one of the rules: absorption, distribution, metabolism, excretion, toxicity properties, and predicted medial lethal dose (LD50) of the compounds were predicted using Swiss-ADME (http://www.swissadme.ch, accessed on 11 October 2021) [106] and PROTOX-II web server (https://tox-new.charite.de/protox_II/, accessed on 14 October 2021) [107].

### 4.4. Ligand Preparation

Ligands were downloaded from PubChem server. Each input file was generated on the ACPYPE server [108]. For this calculation, the semi-empirical charge method AM1-BCC and parameters to reproduce HF/6-31G * RESP charges were selected and implemented in ANTECHAMBER [109]. AMBER to define atom type and zero total net charges were considered. The molecules retrieved are shown in Tables S1–S6, and natural compounds in clinical phase II and III studies were considered as controls, as shown in Table S7, Supplementary Materials [62].

### 4.5. Molecular Dynamics Simulations and Molecular Mechanics Generalized Born Surface Area Calculations

The protein (AT1R) and peptides (tau and amyloid beta) structures were edited in a Text Editor, removing not-essential molecules for calculation. Moreover, the best systems obtained by virtual screening were considered to analyze the coupled systems. Consequently, molecular dynamics (MD) simulations were carried out in GROMACS v. 2020 [110]. We considered an AMBER99SB-ILDN force field, explicit solvent (TIP4P water model), and ions to neutralize the system. The systems were minimized with the steepest descent algorithm for 50,000 steps. Then, the equilibrium MD calculations in the NVT (number of molecules, volume, and temperature constant) canonical ensemble with the V-rescale thermostat at 309.65 K with a calculation of 10 ns were performed.

The final analysis was the MD production without restraint condition in the isobaric-isothermal ensemble with V-rescale thermostat at 309.65 K and Parrinello–Rahman barostat at 1 bar of reference pressure with a trajectory of 100 ns. The thermodynamic parameters of each system were analyzed using the Gromacs tools and the plotting graph realized with Gnuplot v5 package [111].

The molecular mechanics generalized born surface area (MM/GBSA) was used to determine the binding free energy of receptor–ligand using gmx MMPBSA v1.4.1 [112] tool based on MMPBSA.py [113], from AmberTools20 suite. In general, this estimation calculates the free energy difference between the bound and unbound form of the receptor as the following equation:

ΔG_bind_ = G_complex_ − G_protein_ − G_ligand_ (1)

where ΔG_bind_ is the binding free energy and G_complex_ (free energy of complex), G_protein_ (free energy of protein), and G_ligand_ (free energy ligand).

The molecular visualizations of the interaction of the complex receptor–ligand structure and output files were carried out using the VMD v1.9.4 (Visual Molecular Dynamics) software [114], and the 2D diagrams of receptor–ligand were generated and visualized using the Ligplot^+^ software [115].

## 5. Conclusions

This study identified three natural substances with potential effects against tau (rutin), amyloid beta (brassicasterol), and AT1R receptors (floribundic acid) using in silico analysis from the scarce available data and research of Peruvian natural products. The ADME/TOX analysis predicted no toxic effects for these compounds, showing them as possible alternatives for drug formulations. The RMSD simulations and predictions have demonstrated higher docking stabilities for the systems with their optimal ligands compared to their controls.

We observed the potential uses of Peruvian native plants, such as *S. sonchilofolius, L. meyenii,* and *C. lechleri*, against Alzheimer’s disease or dementia-related neurovascular issues.

However, further in vitro and in vivo studies of these compounds are required to confirm these results. In addition, several vegetal sources might be further studied or characterized in order to analyze their potential against more neurovascular or neurodegenerative diseases.

## Figures and Tables

**Table 1:** Values of binding affinity [kcal/mol] of control ligand molecules relative to target peptides obtained during docking stage.

Control	PubChem CID	Compound	Binding Affinity
Tau	936	Nicotinamide	−4.2
	60150609	TRx0237	−3.1
Amyloid beta	91973920	Grapeseed extract	−6.7
	51030870	PQ912	−5.7
	25008296	ALZ 801	−4.2
AT1R	65999	Telmisartan	−8.7
	2541	Candesartan	−7.7
	172198	Angiotensin II	−7.6

**Table 2:** Toxicological assessments, druggability, and drug-likeness properties of the lead compounds predicted.

Control	PubChem CID	Compound	A	B	C	D	E	RO5	Drug-Likeness
Tau	5281327	Brassicasterol	-	-	-	-	890	Yes	−1.2
	15694360	Floribundic acid	-	-	-	+	274	Yes	−4.0
	5280805	Rutin	-	-	-	-	500	No	1.93
	936	Nicotinamide	-	-	-	-	250	Yes	−0.6
Amyloid beta	73062	Kaurenoic acid	+	-	-	-	100	Yes	−6.1
	5281327	Brassicasterol	-	-	-	-	890	Yes	−1.2
	5280805	Rutin	-	-	-	-	500	No	1.93
	91973920	Grapeseed extract	-	-	-	-	2500	No	1.83
AT1R	5280805	Rutin	-	-	-	-	500	No	1.93
	5317667	Glucobrassicin	-	-	-	-	200	Yes	−3.1
	15694360	Floribundic acid	-	-	-	+	274	Yes	−4.0
	65999	Telmisartan	-	-	-	-	500	No	0.95

A = hepatotoxicity, B = carcinogenicity, C = mutagenicity, D = cytotoxicity, E = predicted LD 50 (mg·kg^−1^) (in mice), and RO5 = rule of five by Christopher A. Lipinski.

**Table 3:** Binding free energies estimation for the docked compounds into amyloid beta, tau, and AT1R.

Energy Component	Substance	VDWAALS Kcal·mol^−1^	EEL Kcal·mol^−1^	EGB Kcal·mol^−1^	ESURF Kcal·mol^−1^	ΔGgas Kcal·mol^−1^	ΔGsolv Kcal·.mol^−1^	ΔTOTAL Kcal·mol^−1^
Amyloid beta	Brassicasterol	−43.04 ± 3.4	−4.86 ± 5.9	15.08 ± 5.0	−5.27 ± 0.4	−47.9 ± 6.7	9.81 ± 4.9	−38.08 ± 3.7
	Kaurenoic acid	−28.75 ± 2.7	−12.91 ± 3.9	18.48 ± 2.9	−3.67 ± 0.3	−50.34 ± 5.0	14.8 ± 2.9	−35.54 ± 3.9
	Rutin	−61.97 ± 4.9	−32.69 ± 11.6	54.48 ± 8.7	−7.86 ± 0.6	−94.67 ± 14.4	46.62 ± 8.2	−48.05 ± 7.0
	Grapeseed extract	−37.72 ± 6.7	−51.73 ± 13.6	51.15 ± 8.6	−5.7 ± 0.6	−89.46 ± 14.1	45.45 ± 8.2	−44.00 ± 7.0
Tau	Brassicasterol	−42.49 ± 2.9	−4.87 ± 3.9	14.00 ± 2.7	−4.68 ± 0.3	−47.37 ± 4.5	9.31 ± 2.7	−38.05 ± 3.5
	Floribundic acid	−20.61 ± 6.2	−12.8 ± 10.9	23.9 ± 12.9	−2.70 ± 0.8	−42.55 ± 16.0	21.16 ± 12.1	−21.39 ± 5.2
	Rutin	−33.86 ± 3.9	−36.7 ± 15.8	48.20 ± 11.5	−4.86 ± 0.4	−70.60 ± 15.3	43.34 ± 11.4	−27.25 ± 6.1
	Nicotinamide	−4.04 ± 4.4	−2.86 ± 4.8	5.77 ± 6.5	−0.58 ± 0.6	−6.90 ± 7.9	5.18 ± 6.0	−1.72 ± 2.4
AT1R	Floribundic acid	−59.73 ± 3.0	−27.18 ± 6.6	44.26 ± 3.9	−7.6 ± 0.2	−95.64 ± 6.1	36.66 ± 3.8	−58.98 ± 4.4
	Glucobrassicin	−44.55 ± 2.5	−7.11 ± 7.9	41.77 ± 6.6	−5.64 ± 0.3	−51.66 ± 8.3	36.13 ± 6.6	−15.53 ± 4.5
	Rutin	−61.99 ± 2.7	−7.33 ± 5.3	37.06 ± 4.7	−7.3 ± 0.3	−69.32 ± 5.7	29.76 ± 4.7	−39.56 ± 3.3
	Telmisartan	−55.15 ± 3.0	−10.27 ± 5.8	33.27 ± 5.2	−6.71 ± 0.3	−65.42 ± 6.3	26.56 ± 5.5	−38.87 ± 3.1

## Data Availability

No applicable.

## Supplementary Materials

The following are available online. Table S1: Natural compounds description of *Smallanthus sonchifolius*; Table S2: Natural compounds description of *Lepidium meyenii*; Table S3: Natural compounds description of *Croton lechleri*; Table S4: Natural compounds description of *Uncaria tomentosa*; Table S5: Natural compounds description of *Minthostachys mollis*; Table S6: Natural compounds description of *Physalis peruvianus*; Table S7: Values of binding affinity [kcal/mol] of phytochemical ligand molecules relative to tau peptides obtained during docking stage, Table S8: values of binding affinity [kcal/mol] of phytochemical ligand molecules relative to β-amyloid peptides obtained during docking stage, Table S9: values of binding affinity [kcal/mol] of phytochemical ligand molecules relative to AT2R1 receptor obtained during docking stage.
