# Supplementary material for: In Silico Analysis of Metabolites from Peruvian Native Plants as Potential Therapeutics against Alzheimer’s Disease
Source: Molecules. 2022 Jan 28;27(3):918. doi: 10.3390/molecules27030918 (PMC8838509; doi:10.3390/molecules27030918)
Supplement: Supplementary file 1 [file molecules-27-00918-s001.zip › molecules-1550109-supplementary.pdf]

Table S1. Natural compounds description of *Smallanthus sonchifolius.*

PubChemID	Structure	Name	Part of the plant	MW	cLogP	HBA	HBD	PSA
689043	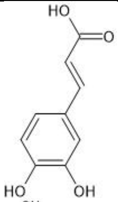	Caffeic acid	Leaves	180.15	0.78	4	3	77.76
1794427	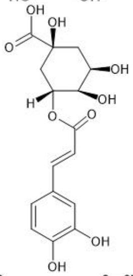	Chlorogenic acid	Leaves	354.31	-0.76	9	6	164.75
445858	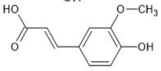	Ferulic acid	Leaves	194.18	1.05	4	2	66.76
131753040	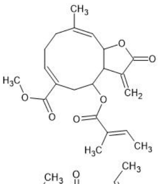	Sonchifolin	Leaves	374.43	4.06	6	0	78.9
101324862	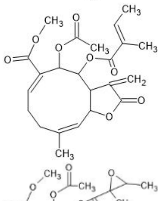	Polymatin B	Leaves	432.46	3.52	8	0	105.2
92043370	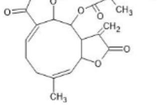	Uvedalin	Leaves	448.46	2.09	9	0	117.73

101250074	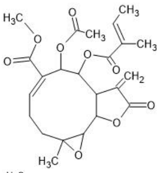	Fluctuanin	Leaves	448.46	2.09	9	0	117.73
73062	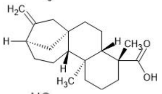	Kaurenoic acid	Leaves	302.45	4.11	2	1	37.3
370	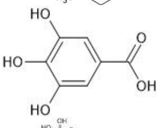	Gallic acid	Leaves	170.12	0.10	5	4	97.99
5280805	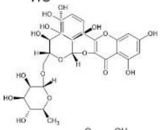	Rutin	Leaves	610.51	-1.25	16	10	265.52
5281672	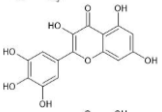	Myricetin	Leaves	318.23	1.14	8	6	147.68
5280863	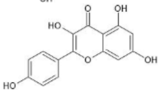	Kaempferol	Leaves	286.23	1.83	6	4	107.22
5280343	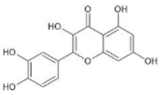	Quercetin	Leaves	302.23	1.49	7	5	127.45

Table S2. Natural compounds description of *Lepidium meyenii.*

Pub-ChemID	Structure	Name	Part of the plant	MW	cLogP	HBA	HBD	PSA
656498	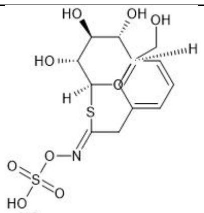	Glucotropaeolin	hypocotyls	409.43	-0.74	10	5	199.79
6602400	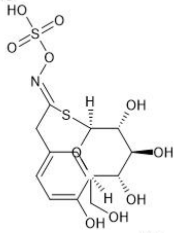	Glucosinalbin	hypocotyls	425.43	-1.09	11	6	220.02
5485207	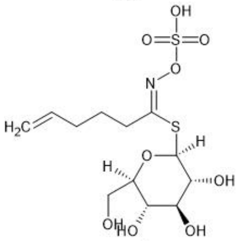	Glucobrassicanapin	hypocotyls	387.42	-0.55	10	5	199.79
5317667	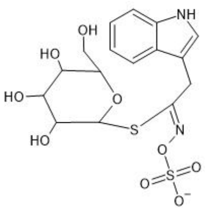	Glucobrassicin	hypocotyls	447.46	-1.95	11	5	218.41

11198769	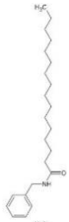	N-Benzylpalmitamide	Leaves	345.56	7.43	2	1	29.1
220495	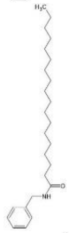	n-Benzyloctadecanamide	Leaves	373.62	8.34	2	1	29.1
68742556	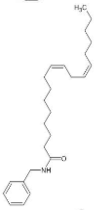	N-Benzyl-linoleamide	Leaves	369.59	7.83	2	1	29.1
68741582	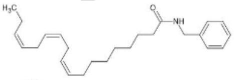	n-Benzyl-(9z,12z,15z)-octa-decatrienamide	Leaves	367.57	7.58	2	1	29.1
5280450	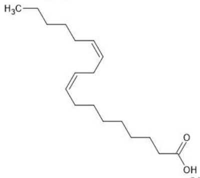	Linoleic acid	Leaves	280.45	6.46	2	1	37.3
445639	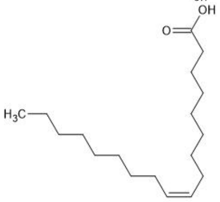	Oleic acid	Leaves	282.46	6.71	2	1	37.3
71386083	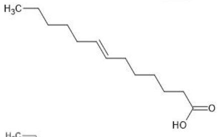	7-Tridecenoic acid	hypocotyls	212.33	4.44	2	1	37.3
71365436	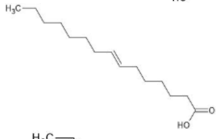	7-pentadecenoic	hypocotyls	240.38	5.35	2	1	37.3
5282748	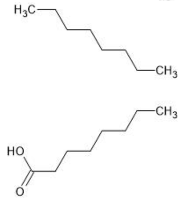	cis-9-Heptadecenoic acid	hypocotyls	268.43	6.26	2	1	37.3
71402235	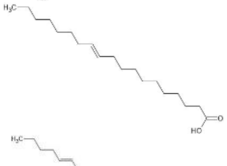	11-Nonadecenoic acid	hypocotyls	296.49	7.17	2	1	37.3
14178780	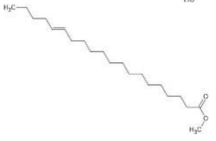	15-Eicosenoic acid	hypocotyls	324.54	8.05	2	0	26.3

222284	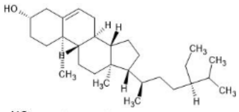	beta-Sitosterol	Leaves	414.71	7.85	1	1	20.23
173183	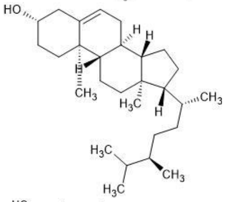	Campesterol	Leaves	400.68	7.40	1	1	20.23
444679	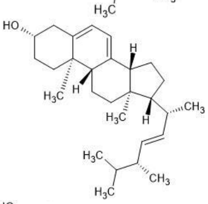	Ergosterol	Leaves	396.65	7.04	1	1	20.23
5281327	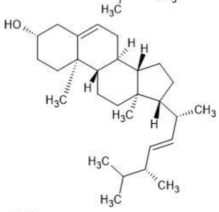	Brassicasterol	Leaves	398.67	7.14	1	1	20.23
636583	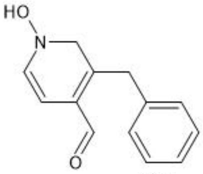	Macaridine	hypocotyls	215.25	0.72	3	1	40.54
25310	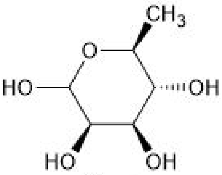	L-Rhamnose	hypocotyls	164.15	-1.69	5	4	90.15
439195	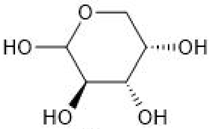	L-Arabinose	hypocotyls	150.12	-2.01	5	4	90.15
5793	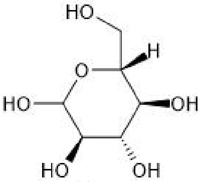	D-Glucose	hypocotyls	180.15	-2.62	6	5	110.38
6036	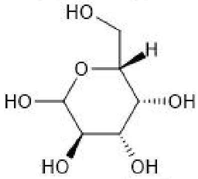	D-Galactose	hypocotyls	180.15	-2.62	6	5	110.38
8794	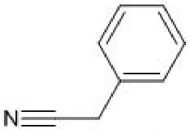	Phenylacetonitrile	Leaves	117.15	1.66	1	0	23.79
240	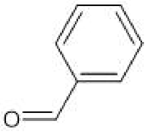	Benzaldehyde	Leaves	106.12	1.59	1	0	17.07

Table S3. Natural compounds description of *Croton lechleri.*

PubChemID	Structure	Name	Part of the plant	MW	cLogP	HBA	HBD	PSA
15694360	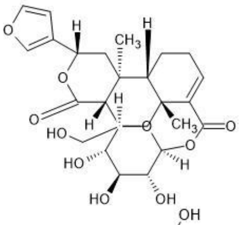	Floribundic acid glucoside	Sap	506.54	0.54	10	4	155.89
102237419	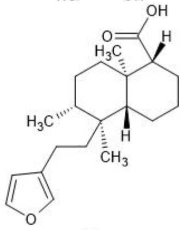	Crolechinic acid	Bark	318.45	4.20	3	1	50.44
101926685	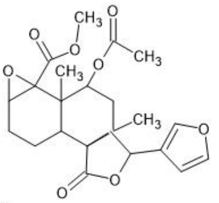	Korberin	Bark	432.46	1.42	8	0	104.57
124426	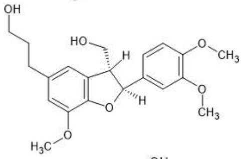	3',4-O-Dimethylcedrusin	Sap	374.43	3.23	6	2	77.38
134820409	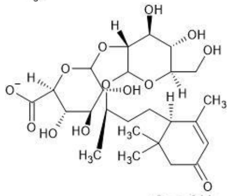	Blumenol	Bark	547.57	-3.37	13	6	215.5
69301	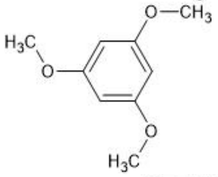	1,3,5-Trimethoxybenzene	Bark	168.19	1.44	3	0	27.69
88563	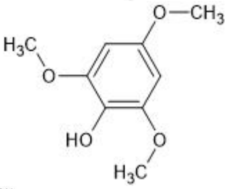	2,4,6-Trimethoxyphenol	Bark	184.19	1.10	4	1	47.92
44149614	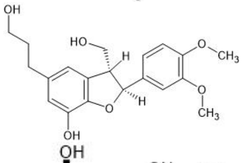	4-O-Methylcedrusin	Bark	360.40	2.95	6	3	88.38
21630916	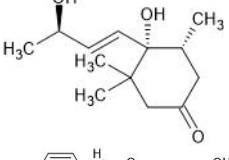	4,5-Dihydroblumenol A	Bark	226.31	1.74	3	2	57.53
9064	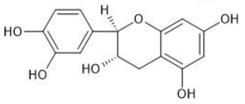	Cianidanol	Sap	290.27	1.50	6	5	110.38

72276	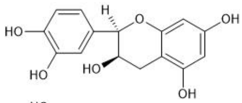	(-)-Epicatechin	Sap	290.27	1.50	6	5	110.38
65084	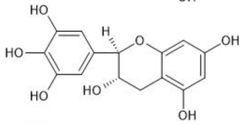	(+)-Gallocatechin	Sap	306.26	1.16	7	6	130.61
72277	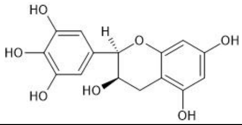	Epigallocatechin	Sap	306.26	1.16	7	6	130.61

Table S4. Natural compounds description of *Uncaria tomentosa.*

Pub-ChemID	Structure	Name	Part of the plant	MW	cLogP	HBA	HBD	PSA
9885603	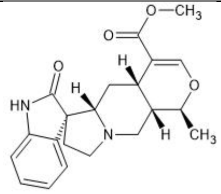	Isopteropodine	Bark	368.43	0.84	6	1	67.87
168985	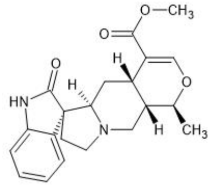	Speciophylline	Leaves	368.43	0.84	6	1	67.87
12304288	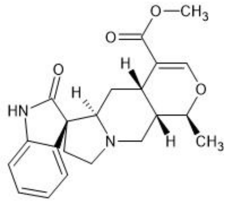	Uncarine F	Bark	368.43	0.84	6	1	67.87
94160	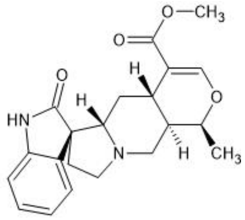	Mitraphylline	Leaves	368.43	0.84	6	1	67.87
11726520	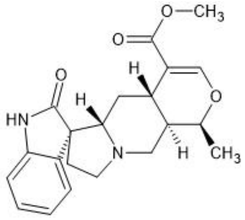	Isomitraphylline	Leaves	368.43	0.84	6	1	67.87
5281408	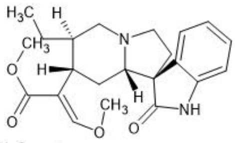	Rhynchophylline	Leaves	384.47	1.88	6	1	67.87
3037048	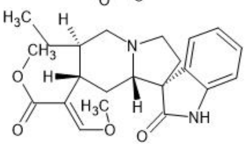	Isorhynchophylline	Leaves	384.47	1.88	6	1	67.87

44568160	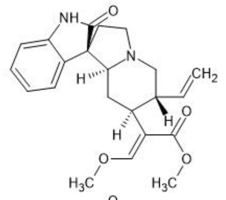	Corynoxeine	Bark	382.45	1.70	6	1	67.87
3037448	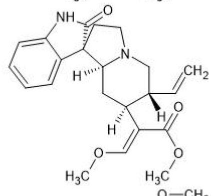	Isocorynoxeine	Bark	382.45	1.702	6	1	67.87
1268096	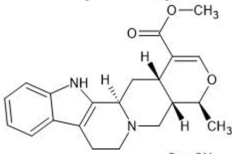	Akuammigine	Bark	352.43	2.264	5	1	54.56
72340	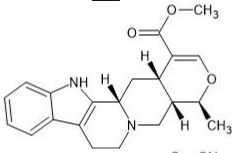	Tetrahydroalstonine	Bark	352.4	2.26	5	1	54.56
179461	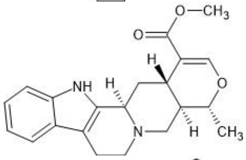	19-Epi-3-Isoajmali-cine	Bark	352.43	2.26	5	1	54.56
3037884	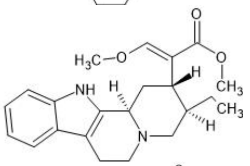	Hirsutine	Roots	368.4	3.31	5	1	54.56
3039336	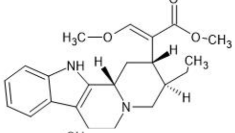	Dihydrocorynanthe-ine	Bark	368.47	3.31	5	1	54.56
3037151	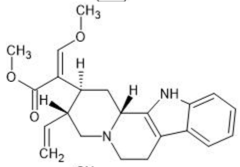	Hirsuteine	Bark	366.45	3.12	5	1	54.56
3037997	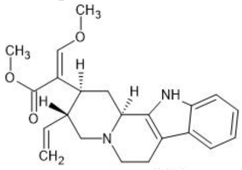	Corynantheine	Bark	366.45	3.12	5	1	54.56
439746	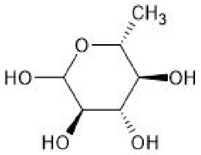	Quinovose	Bark	164.15	-1.69	5	4	90.15
17106	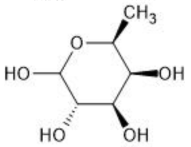	Fucose	Bark	164.1	-1.69	5	4	90.15
25310	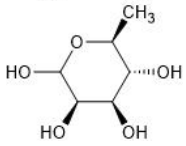	Rhamnose	Bark	164.15	-1.69	5	4	90.15

5793	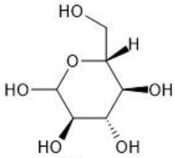	Glucose	Bark	180.15	-2.62	6	5	110.38
6036	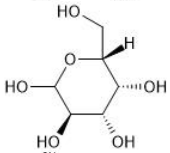	Galactose	Bark	180.15	-2.62	6	5	110.38
246983	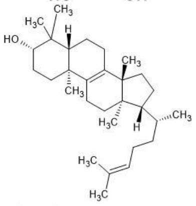	Lanosterol	Leaves	426.72	8.42	1	1	20.23
5280794	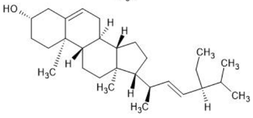	Stigmasterol	Leaves	412.69	7.60	1	1	20.23
12795736	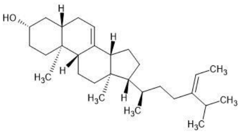	delta7-Avenasterol	Bark	412.69	7.99	1	1	20.23

Table S5. Natural compounds description of *Minthostachys mollis.*

PubChemID	Structure	Name	Part of the plant	MW	cLogP	HBA	HDA	PSA
1254	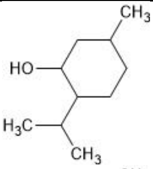	Menthol	Leaves	156.26	2.4112	1	1	20.23
22311	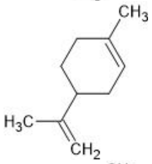	Limonene	Leaves	136.23	3.3614	0	0	0
10364	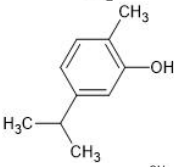	Carvacrol	Leaves	150.22	2.8448	1	1	20.23
5284507	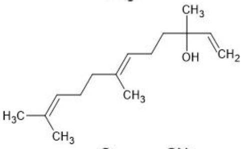	Nerolidol	Leaves	222.37	5.4029	1	1	20.23
7439	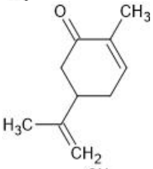	Carvone	Leaves	150.22	2.653	1	0	17.07
5281515	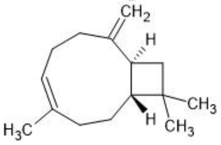	Caryophyllene	Leaves	204.35	5.4861	0	0	0

6549	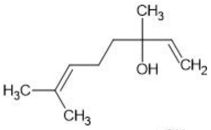	Linalool	Leaves	154.25	3.2311	1	1	20.23
9548705	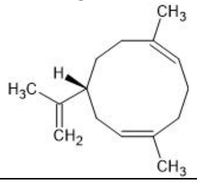	GermacreneÂ	Leaves	204.35	6.346	0	0	0

Table S6. Natural compounds description of *Physalis peruvianus.*

PubChemID	Structure	Name	Part of the plant	MW	cLogP	HBA	HDA	PSA
246983	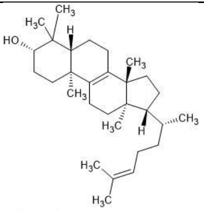	Lanosterol	Fruit	426.72	8.42	1	1	20.23
5280794	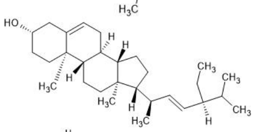	Stigmasterol	Fruit	412.69	7.60	1	1	20.23
12795736	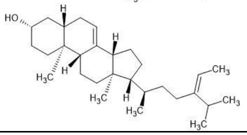	delta7-Avenasterol	Fruit	412.69	7.99	1	1	20.23

Table S7. Values of binding affinity [kcal/mol] of phytochemical ligand molecules relative to Tau peptides obtained during docking stage

Name	Pubchem CID	Binding Affinity (Tau)
Brassicasterol	5281327	-8.1
Floribundic acid	15694360	-8.1
Rutin	5280805	-7.6
(+)-Gallocatechin	65084	-7.4
Chlorogenic acid	1794427	-7.4
Epigallocatechin	72277	-7.4
Isopteropodine	9885603	-7.4
Mitraphylline	94160	-7.4
(-)-Epicatechin	72276	-7.3
Isomitraphylline	11726520	-7.3
Quercetin	5280343	-7.3
Speciophylline	168985	-7.3
Stigmasterol	5280794	-7.3
Uncarine F	12304288	-7.3
n-Benzyloctadecanamide	220495	-7.3
Blumenol	134820409	-7.2
Cianidanol	9064	-7.2
Myricetin	5281672	-7.2
delta7-Avenasterol	12795736	-7.2

Campesterol	173183	-7.1
Corynantheine	3037997	-7.1
beta-Sitosterol	222284	-7.1
Kaempferol	5280863	-6.9
Dihydrocorynantheine	3039336	-6.8
Hirsuteine	3037151	-6.8
Hirsutine	3037884	-6.8
N-Benzylpalmitamide	11198769	-6.8
Nerolidol	5284507	-6.8
4-O-Methylcedrusin	44149614	-6.7
3',4-O-Dimethylcedrusin	124426	-6.5
Glucobrassicin	5317667	-6.5
Korberin	101926685	-6.5
N-Benzyl-linoleamide	68742556	-6.5
Sonchifolin	131753040	-6.5
Uvedalin	92043370	-6.5
Rhynchophylline	5281408	-6.4
15-Eicosenoic acid	14178780	-6.3
Corynoxeine	44568160	-6.3
Crolechinic acid	102237419	-6.3
Isocorynoxeine	3037448	-6.3
Isorhynchophylline	3037048	-6.3
Fluctuanin	101250074	-6.2
Glucotropaeolin	656498	-6.2
n-Benzyl-(9z,12z,15z)-octadecatrien-amide	68741582	-6.2
Glucosinalbin	6602400	-6.1
GermacreneÂ	9548705	-5.9
cis-9-Heptadecenoic acid	5282748	-5.9
Ferulic acid	445858	-5.8
Polymatin B	101324862	-5.7
7-Tridecenoic acid	71386083	-5.6
Caffeic acid	689043	-5.6
Glucobrassicanapin	5485207	-5.5
Macaridine	636583	-5.5
Linalool	6549	-5.4
Caryophyllene	5281515	-5.3
19-Epi-3-Isoajmalicine	179461	-5.3
Akuammigine	1268096	-5.3
Carvacrol	10364	-5.2
Glucose	5793	-5.2
2,4,6-Trimethoxyphenol	88563	-5.1
D-Galactose	6036	-5.1
D-Glucose	5793	-5.1
Galactose	6036	-5.1
Tetrahydroalstonine	72340	-5.0
Linoleic acid	5280450	-5.0
Carvone	7439	-4.9

Fucose	17106	-4.9
Gallic acid	370	-4.9
L-Rhamnose	25310	-4.9
Quinovose	439746	-4.9
Rhamnose	25310	-4.9
1, 3,5-Trimethoxybensene	69301	-4.8
7-pentadecenoic	71365436	-4.8
Limonene	22311	-4.8
L-Arabinose	439195	-4.6
Menthol	1254	-4.5
Phenylacetonitrile	8794	-4.5
Ergosterol	444679	-4.2
Kaurenoic acid	73062	-4.2
Oleic acid	445639	-4.2
Lanosterol	246983	-4.0
Benzaldehyde	240	-4.0

Table S8. Values of binding affinity [kcal/mol] of phytochemical ligand molecules relative to β-amyloid peptides obtained during docking stage.

Name	PubChem CID	Binding Affinity (Amyloid beta)
Kaurenoic acid	73062	-6.3
Brassicasterol	5281327	-6.3
Rutin	5280805	-6.2
beta-Sitosterol	222284	-6.1
Stigmasterol	5280794	-6
Campesterol	173183	-5.9
Floribundic acid glucoside	15694360	-5.9
Akuammigine	1268096	-5.9
19-Epi-3-Isoajmalicine	179461	-5.9
Ergosterol	444679	-5.8
Tetrahydroalstonine	72340	-5.8
delta7-Avenasterol	12795736	-5.7
Dihydrocorynantheine	3039336	-5.6
Hirsuteine	3037151	-5.6
Corynantheine	3037997	-5.6
Lanosterol	246983	-5.6
Kaempferol	5280863	-5.5
Isopteropodine	9885603	-5.4
Speciophylline	168985	-5.4
Uncarine F	12304288	-5.4
Mitraphylline	94160	-5.4
Isomitraphylline	11726520	-5.4
Quercetin	5280343	-5.3
4-O-Methylcedrusin	44149614	-5.3
Myricetin	5281672	-5.2
Cianidanol	9064	-5.2

(-)-Epicatechin	72276	-5.2
Hirsutine	3037884	-5.2
Chlorogenic acid	1794427	-5.1
Fluctuanin	101250074	-5.1
Glucobrassicin	5317667	-5.1
Crolechinic acid	102237419	-5.1
(+)-Gallocatechin	65084	-5.1
Epigallocatechin	72277	-5.1
Korberin	101926685	-5
3',4-O-Dimethylcedrusin	124426	-5
Sonchifolin	131753040	-4.9
Isocorynoxeine	3037448	-4.9
Polymatin B	101324862	-4.8
n-Benzyloctadecanamide	220495	-4.8
Blumenol	134820409	-4.8
Rhynchophylline	5281408	-4.8
Isorhynchophylline	3037048	-4.8
Germacrene	9548705	-4.8
Glucotropaeolin	656498	-4.7
Corynoxeine	44568160	-4.7
Nerolidol	5284507	-4.7
Uvedalin	92043370	-4.6
Linoleic acid	5280450	-4.6
Macaridine	636583	-4.6
Caryophyllene	5281515	-4.6
Glucosinalbin	6602400	-4.5
cis-9-Heptadecenoic acid	5282748	-4.5
Glucobrassicanapin	5485207	-4.4
Oleic acid	445639	-4.4
Menthol	1254	-4.4
Carvacrol	10364	-4.4
Carvone	7439	-4.4
Caffeic acid	689043	-4.3
11-Nonadecenoic acid	71402235	-4.2
4,5-Dihydroblumenol A	21630916	-4.2
Limonene	22311	-4.2
Ferulic acid	445858	-4.1
7-Tridecenoic acid	71386083	-4.1
7-pentadecenoic	71365436	-4.1
N-Benzyl-linoleamide	68742556	-4
Linalool	6549	-4
Phenylacetonitrile	8794	-3.9
Gallic acid	370	-3.8
D-Glucose	5793	-3.8
D-Galactose	6036	-3.8
2,4,6-Trimethoxyphenol	88563	-3.8
Glucose	5793	-3.8

Galactose	6036	-3.8
Benzaldehyde	240	-3.7
L-Rhamnose	25310	-3.6
Quinovose	439746	-3.6
Fucose	17106	-3.6
Rhamnose	25310	-3.6
n-Benzyl-(9z,12z,15z)-octadecatriena-mide	68741582	-3.5
L-Arabinose	439195	-3.4
1,3,5-Trimethoxybenzene	69301	-3.4
15-Eicosenoic acid	14178780	-3.2
N-Benzylpalmitamide	11198769	-3

Table S9. Values of binding affinity [kcal/mol] of phytochemical ligand molecules relative to AT2R1 receptor obtained during docking stage.

**Name**	**PubChem CID**	**Binding Affinity (AT2R1)**
Rutin	5280805	-7.5
Glucobrassicin	5317667	-7.3
Floribundic acid glucoside	15694360	-7.3
Lanosterol	246983	-7.2
Ergosterol	444679	-7.1
Akuammigine	1268096	-7.1
Tetrahydroalstonine	72340	-7.1
Chlorogenic acid	1794427	-7
Campesterol	173183	-6.9
19-Epi-3-Isoajmalicine	179461	-6.9
Kaurenoic acid	73062	-6.8
Korberin	101926685	-6.8
Isopteropodine	9885603	-6.8
Mitraphylline	94160	-6.8
Hirsuteine	3037151	-6.8
Glucosinalbin	6602400	-6.7
Brassicasterol	5281327	-6.7
Blumenol	134820409	-6.7
Speciophylline	168985	-6.7
Uncarine F	12304288	-6.7
Isomitraphylline	11726520	-6.7
Hirsutine	3037884	-6.7
Stigmasterol	5280794	-6.7
Myricetin	5281672	-6.6
Kaempferol	5280863	-6.6
Sonchifolin	131753040	-6.5
Fluctuanin	101250074	-6.5
Isorhynchophylline	3037048	-6.5
Quercetin	5280343	-6.4
Glucotropaeolin	656498	-6.4
(-)-Epicatechin	72276	-6.4

Epigallocatechin	72277	-6.4
Corynoxeine	44568160	-6.4
Isocorynoxeine	3037448	-6.4
Crolechinic acid	102237419	-6.3
(+)-Gallocatechin	65084	-6.3
delta7-Avenasterol	12795736	-6.3
Caryophyllene	5281515	-6.3
Uvedalin	92043370	-6.2
Cianidanol	9064	-6.2
Rhynchophylline	5281408	-6.2
Dihydrocorynantheine	3039336	-6.2
Corynantheine	3037997	-6.2
4-O-Methylcedrusin	44149614	-6.1
Limonene	22311	-6.1
Germacrene	9548705	-6.1
beta-Sitosterol	222284	-6
Carvacrol	10364	-6
Carvone	7439	-6
Polymatin B	101324862	-5.9
Glucobrassicanapin	5485207	-5.9
3',4-O-Dimethylcedrusin	124426	-5.9
Ferulic acid	445858	-5.8
Macaridine	636583	-5.8
Caffeic acid	689043	-5.7
Menthol	1254	-5.6
Gallic acid	370	-5.3
n-Benzyl-(9z,12z,15z)-octadeca-trienamide	68741582	-5.3
Benzaldehyde	240	-5.3
N-Benzyl-linoleamide	68742556	-5.2
11-Nonadecenoic acid	71402235	-5.1
Linoleic acid	5280450	-5
1,3,5-Trimethoxybenzene	69301	-5
4,5-Dihydroblumenol A	21630916	-5
Nerolidol	5284507	-5
n-Benzyloctadecanamide	220495	-4.9
15-Eicosenoic acid	14178780	-4.8
2,4,6-Trimethoxyphenol	88563	-4.8
7-pentadecenoic	71365436	-4.6
Linalool	6549	-4.6
Oleic acid	445639	-4.5
7-Tridecenoic acid	71386083	-4.5
L-Rhamnose	25310	-4.5
D-Glucose	5793	-4.5
D-Galactose	6036	-4.5
Phenylacetonitrile	8794	-4.5
Quinovose	439746	-4.5
Fucose	17106	-4.5

Rhamnose	25310	-4.5
Glucose	5793	-4.5
Galactose	6036	-4.5
cis-9-Heptadecenoic acid	5282748	-4.4
L-Arabinose	439195	-4.3
N-Benzylpalmitamide	11198769	-4.2
